# Activation of ERK–Drp1 signaling promotes hypoxia‐induced Aβ accumulation by upregulating mitochondrial fission and BACE1 activity

**DOI:** 10.1002/2211-5463.13273

**Published:** 2021-08-27

**Authors:** Yuan Yuan, Jingjiong Chen, Xuhua Ge, Jiangshan Deng, Xiaofeng Xu, Yuwu Zhao, Hongmei Wang

**Affiliations:** ^1^ Department of Neurology Shanghai Jiao Tong University Affiliated Sixth People's Hospital China; ^2^ Department of General Medicine Yangpu Hospital Tongji University School of Medicine Shanghai China

**Keywords:** Alzheimer's disease, BACE1, ERK, hypoxia, mitochondrial fission

## Abstract

Hypoxia is a risk factor for Alzheimer's disease (AD). Besides, mitochondrial fission is increased in response to hypoxia. In this study, we sought to investigate whether hypoxia‐induced mitochondrial fission plays a critical role in regulating amyloid‐β (Aβ) production. Hypoxia significantly activated extracellular signal‐regulated kinase (ERK), increased phosphorylation of dynamin‐related protein 1 (Drp1) at serine 616, and decreased phosphorylation of Drp1 at serine 637. Importantly, hypoxia triggered mitochondrial dysfunction, elevated β‐secretase 1 (BACE1) and γ‐secretase activities, and promoted Aβ accumulation in HEK293 cells transfected with β‐amyloid precursor protein (APP) plasmid harboring the Swedish and Indiana familial Alzheimer's disease mutations (APPSwe/Ind HEK293 cells). Then, we investigated whether the ERK inhibitor PD325901 and Drp1 inhibitor mitochondrial division inhibitor‐1 (Mdivi‐1) would attenuate hypoxia‐induced mitochondrial fission and Aβ generation in APPSwe/Ind HEK293 cells. PD325901 and Mdivi‐1 inhibited phosphorylation of Drp1 at serine 616, resulting in reduced mitochondrial fission under hypoxia. Furthermore, hypoxia‐induced mitochondrial dysfunction, BACE1 activation, and Aβ accumulation were downregulated by PD325901 and Mdivi‐1. Our data demonstrate that hypoxia induces mitochondrial fission, impairs mitochondrial function, and facilitates Aβ generation. The ERK–Drp1 signaling pathway is partly involved in the hypoxia‐induced Aβ generation by regulating mitochondrial fission and BACE1 activity. Therefore, inhibition of hypoxia‐induced mitochondrial fission may prevent or slow the progression of AD.

## Graphical abstract

Hypoxia is a risk factor for Alzheimer's disease. However, whether hypoxia‐induced mitochondrial fission plays a critical role in the regulation of amyloid‐β (Aβ) accumulation has not yet been investigated. In the current study, we show that the ERK/Drp1 signaling pathway is partly involved in regulating mitochondrial fission and Aβ generation under hypoxia.

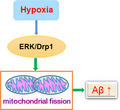

**Abbreviations**
∆ψm: mitochondrial membrane potential
AD: Alzheimer's disease
APP: β‐amyloid precursor protein
ATP: adenosine triphosphate
Aβ: amyloid‐β
BACE1: β‐site APP cleaving enzyme 1
DCPIP: dichlorophenolindophenol
DMEM: Dulbecco's modified eagle medium
Drp1: dynamin‐related protein 1
ERK: extracellular signal‐regulated kinase
JC‐1: 6′‐tetrachloro‐1,1′,3,3′‐tetraethylbenzimidazolcarbocyanine iodide
LDH: lactate dehydrogenase
MDA: malondialdehyde
Mdivi-1: mitochondrial division inhibitor‐1
Mfn1: mitofusin 1
Mfn2: mitofusin 2
MTT: 3-(4,5-dimethylthiazol-2-yl)-2,5-diphenyltetrazolium bromide
ROS: reactive oxygen species
TBA: thiobarbituric acid

Alzheimer's disease (AD) is the most common cause of dementia characterized by a progressive decline of memory and other cognitive functions. The two primary pathological hallmarks of AD are extracellular amyloid plaques formed by the amyloid‐β (Aβ) peptide and intracellular neurofibrillary tangles, composed of hyperphosphorylated and misfolded tau protein. Aβ peptide is derived from the amyloid precursor protein (APP) through sequential proteolytic cleavages by β‐ and γ‐secretase.

Alzheimer's disease is also associated with other features, including mitochondrial dysfunction, oxidative stress, synaptic failure, and neuroinflammation. Mitochondrial dysfunction is an early pathological feature of AD and may play an important role in the pathogenesis of disease. Mitochondria produce most of the adenosine triphosphate (ATP) and are vital for cellular energy supply. Besides, mitochondria are considered as a major source of reactive oxygen species (ROS) in cells [1]. Therefore, mitochondria play a critical role in controlling overall energy homeostasis and regulating oxidative stress. Mitochondria are highly dynamic organelles and undergo continuous fission and fusion to maintain neuronal function. Dynamin‐related protein 1 (Drp1) is a cytosolic GTPase that mediates mitochondrial fission, which is involved in various cellular functions. Phosphorylation of Drp1 at serine 616 (Ser616) promotes Drp1‐mediated mitochondrial fission, whereas phosphorylation of Drp1 at serine 637 (Ser616) inhibits Drp1 enzyme activity, resulting in reduced mitochondrial fission [2, 3]. However, mitochondrial fusion is controlled by optic atrophy 1 (Opa1), mitofusin 1 (Mfn1), and mitofusin 2 (Mfn2) [4, 5]. Importantly, elevated mitochondrial fission and decreased mitochondrial fusion have been found in AD [6, 7, 8]. Consistent with these studies, increased expression of Drp1 along with decreased levels of Mfn1 and Mfn2 is confirmed in frontal cortex from AD patients [9]. Excessive mitochondrial fission is tightly linked to mitochondrial fragmentation, leading to decreased ATP production and increased ROS production, which exacerbate mitochondrial dysfunction associated with neurodegenerative diseases such as Alzheimer's disease, Huntington's disease, and Parkinson's disease. In addition, mitochondrial fragmentation contributes to aberrant synaptic structure and function associated with AD pathogenesis [10]. Therefore, inhibition of mitochondrial fission may be a potential therapeutic target for AD.

It is well established that hypoxia impairs cognitive functions associated with mild cognitive impairment or dementia [11, 12]. Increasing evidence suggests that cerebral ischemia, cerebral vascular atherosclerosis, and hypoperfusion contribute to AD pathogenesis by facilitating Aβ deposition. In addition, hypoxia increases β‐ and γ‐secretase activities and enhances Aβ deposition [11, 13, 14, 15, 16, 17]. These results confirm that hypoxia and ischemia are linked to increased risk of AD. Recent studies reveal that hypoxic or ischemic insults are associated with increased mitochondrial fission [18, 19]. Nevertheless, whether hypoxia‐induced mitochondrial fission plays a critical role in the regulation of Aβ accumulation has not yet been investigated.

Extracellular signal‐regulated kinase (ERK) belongs to the family of mitogen‐activated protein kinases and is involved in the pathogenesis of AD [20, 21, 22]. Enhanced phospho‐ERK (p‐ERK) is observed in the brains of AD patients and AD mouse models [23, 24]. Recent studies reveal that the activation of ERK induces mitochondrial fission through direct phosphorylation of Drp1 at serine 616 [25, 26]. In addition, ERK has been reported to be activated in response to ischemia or hypoxia [27, 28]. However, whether ERK/Drp1 signaling is involved in regulating hypoxia‐induced mitochondrial fission and Aβ accumulation has not been examined.

The aim of this study was to elucidate the role of ERK/Drp1 pathway in the regulation of hypoxia‐induced Aβ generation. We found that hypoxia promotes mitochondrial fission, impairs mitochondrial function, elevates Aβ_42_ production, and enhances cell death in APPSwe/Ind HEK293 cells. However, ERK inhibitor PD325901 and mitochondrial division inhibitor‐1 (Mdivi‐1), a selective inhibitor of the Drp1, protect APPSwe/Ind HEK293 cells against hypoxia‐induced mitochondrial fission. Importantly, PD325901 and Mdivi‐1 obviously ameliorated β‐site APP cleaving enzyme 1 (β‐secretase 1, BACE1) activity, leading to reduced Aβ_42_ generation under hypoxia. These data demonstrate that PD325901 and Mdivi‐1 inhibit hypoxia‐induced mitochondrial fission and Aβ accumulation in APPSwe/Ind HEK293 cells. Furthermore, PD325901 and Mdivi‐1 significantly attenuated hypoxia‐induced cell death, indicating that PD325901 and Mdivi‐1 exert protective effects in APPSwe/Ind HEK293 cells exposed to hypoxia.

Taken together, our results show that ERK/Drp1 signaling pathway is partly involved in regulating mitochondrial fission and Aβ generation under hypoxia and may offer a feasible therapeutic target for dementia associated with hypoxia, including AD.

## Materials and methods

### Antibodies and reagents

The pCAX APP Swe/Ind plasmid was from Addgene, Beijing, China (plasmid # 30145). Dulbecco's modified eagle medium (DMEM), FBS, Lipofectamine 2000, 3‐(4,5‐dimethylthiazol‐2‐yl)‐2,5‐diphenyltetrazolium bromide (MTT), and Aβ_42_ ELISA kit were purchased from Invitrogen (Carlsbad, CA, USA). Mdivi‐1, lactate dehydrogenase (LDH) activity assay kit, and anti‐β‐actin primary antibody were purchased from Sigma‐Aldrich (St. Louis, MO, USA). Caspase‐3 colorimetric assay kit was purchased from BioVision (Milpitas, CA, USA). β‐Secretase and γ‐secretase activity assay kits were purchased from R&D Systems (Minneapolis, MN, USA). Anti‐BACE1, anti‐phospho‐Drp1 (Ser616), anti‐phospho‐Drp1 (Ser637), anti‐ERK1/2, and anti‐phospho‐ERK1/2 (Thr202/Tyr204) were obtained from Cell Signaling Technology (Beverly, MA, USA). Anti‐Drp1, secondary anti‐mouse and anti‐rabbit antibodies were purchased from Santa Cruz Biotechnology (Santa Cruz, CA, USA). Anti‐Mfn1, anti‐Mfn2, and anti‐HIF‐1α were purchased from Abcam (Cambridge, MA, USA). CM‐H2DCFDA, 5, 5′, 6, MitoSOX™ Red, 6′‐tetrachloro‐1,1′,3,3′‐tetraethylbenzimidazolcarbocyanine iodide (JC‐1), and MitoTracker Red were purchased from Molecular Probes (Eugene, OR, USA). ERK1/2 inhibitor PD325901 was provided by MedChemExpress (Monmouth Junction, NJ, USA). Enhanced chemiluminescence reagents were purchased from Amersham Pharmacia Biotech (Piscataway, NJ, USA).

### Cell culture and cell transfection

HEK293 cells were obtained from the Cell Bank of the Chinese Academy of Sciences (Shanghai, China). Cells were cultured at 37 °C in DMEM supplemented with 10% FBS. Cells were transfected with APPSwe/Ind plasmid [29] using Lipofectamine 2000 following the manufacturer's instructions. Experiments were performed 23–48 h after transfection.

### Hypoxia exposure

For hypoxia, APPSwe/Ind‐transfected HEK293 cells were placed in a hypoxic incubator filled with mixed gas containing 1% O_2_ and incubated at 37 °C for 0, 2, 8, and 24 h.

### Mitochondrial respiratory chain complex activities

Mitochondrial fractions were obtained using a mitochondria isolation kit (Beyotime, Jiangsu, China) according to manufacturer's instructions. Activities of complexes I, II, III, and IV were determined following previous published methods. Mitochondrial complex I activity was assayed as the rotenone sensitive rate of NADH oxidation at 340 nm [30, 31]. The reaction mix contains 0.5 mm KCN, 2.4 μg·mL^−1^ antimycin A, 25 mm KH_2_PO_4_, 2 mg·mL^−1^ BSA, 1 mm NaN_3_, 0.24 mm CoQ_1_, 5 mm MgCl_2_, and 0.15 mm NADH. The mitochondrial complex I inhibitor rotenone (5 µm) was added, and the inhibited rate was measured. Mitochondrial complex II activity was determined by measuring the reduction in dichlorophenolindophenol (DCPIP) [31, 32]. The reaction mix contains 5 mm KCN, 4 mm antimycin A, 5 μm rotenone, 20 mm succinate, 50 mm KH_2_PO_4_, 2 mg·mL^−1^ BSA, and 50 μm DCPIP. The absorbance was monitored at 600 nm. Mitochondrial complex III activity was monitored as cytochrome *c* reduction at 550 nm [30, 33]. The reaction mix contains 0.5 mm KCN, 5 mm NADH, 5 μm rotenone, 50 mm KH_2_PO_4_, 2 mg·mL^−1^ BSA, 1 mm NaN_3_, and 0.12 mm cytochrome *c*. Mitochondrial complex IV activity was assessed by measuring the oxidation of reduced cytochrome *c* as previously described [34]. The reaction mix contains 25 mm KH_2_PO_4_, 0.45 mm dodecyl maltoside, and 15 μm reduced cytochrome *c*. The absorbance was monitored at 550 nm. Relative activity of each complex was presented as percentage activity compared to the control.

### ATP levels

ATP levels were measured using an ATP bioluminescence assay kit (Promega, Madison, WI, USA) following the manufacturer's manual.

### JC‐1 fluorescence analysis

Mitochondrial membrane potential was assessed using JC‐1 dye. Cells were incubated with 10 μg·mL^−1^ JC‐1 for 15 min at 37 °C, and images were taken with a fluorescence microscope. Mitochondrial depolarization was monitored by a reduction in the red (aggregates)/green (monomers) fluorescence intensity ratio.

### Measurement of intracellular ROS levels

Briefly, cells were incubated with 10 µm CM‐H2DCFDA for 30 min at 37 °C. Intracellular ROS levels were evaluated using a fluorescence spectrometer. Each measurement was performed in triplicate.

### Measurement of mitochondrial ROS levels

Cells were incubated with 5 μm MitoSOX™ Red for 10 min at 37 °C. Mitochondrial ROS levels were analyzed using a fluorescence spectrometer. Each measurement was performed in triplicate.

### Malondialdehyde levels

Malondialdehyde (MDA) levels were detected using a commercial assay kit (Jiancheng Bioengineering Institute, Nanjing, China) according to manufacturer's instructions. MDA reacts with thiobarbituric acid (TBA) to form MDA‐TBA2 (a red‐colored adduct) that can be assessed spectrophotometrically at 532 nm. The reaction mixtures were incubated in a water heater at 95 °C for 40 min. Upon cooling, the tubes were centrifuged at 2465 ***g*** for 10 min and the supernatant was analyzed for MDA.

### MTT assay

Cell viability was performed using MTT assay as previously described [34]. After various treatments, cells were incubated with 0.5 mg·mL^−1^ MTT for 4 h at 37 °C. Media was then carefully removed, and formazan crystals were dissolved in dimethyl sulfoxide. Absorbance was measured at 570 nm using a microplate reader. Each measurement was performed at least three times.

### Lactate dehydrogenase assay

Lactate dehydrogenase activity was determined using the commercial LDH assay kit (Sigma‐Aldrich) following manufacturer's protocols.

### Measurement of caspase‐3 activity

The caspase‐3 activity was quantified by caspase‐3 colorimetric assay kit (BioVision) according to the manufacturer's instructions. The activity of caspase‐3 was measured by the cleavage of the colorimetric substrate. Absorbance was measured at 405 nm using a microplate reader.

### β‐Secretase activity and γ‐secretase activity

β‐Secretase activity and γ‐secretase activity were detected using β‐secretase activity and γ‐secretase activity assay kits (R&D Systems) according to manufacturer's protocols.

### Aβ ELISA

Human Aβ_42_ levels were measured using the commercial sandwich ELISA kit (Invitrogen) according to the manufacturer's instructions.

### Immunofluorescence

Cells were cultured on coverslips. Following various treatments, cells were fixed with 4% paraformaldehyde in PBS for 20 min and permeabilized with 0.1% Triton X‐100 in PBS for 30 min. Cells were blocked in blocking buffer for 1 h at room temperature and then incubated with primary antibodies against p‐ERK or BACE1 overnight at 4 °C. After incubation, cells were washed with PBS and incubated with Alexa 488 or Alexa 594 secondary antibodies for 1 h at room temperature. Nuclei were stained with DAPI, and images were captured with a fluorescence microscope.

### Western blot analysis

Samples containing equal amounts of protein were separated on 10–12% SDS/PAGE gels, transferred to PVDF membranes, and then incubated with a blocking buffer (5% skim milk in TBST) for 1 h at room temperature. The PVDF membranes were probed with the primary antibodies overnight at 4 °C. The blots were washed in TBST buffer and then incubated with appropriate HRP‐conjugated secondary antibodies for 1 h at room temperature. The membrane was stripped and then reprobed with a second primary antibody. Proteins were visualized with enhanced chemiluminescence reagents.

### Measurement of mitochondria morphology

Mitochondrial morphology was visualized and analyzed using MitoTracker Red staining as previously described [35, 36]. Mitochondrial morphology was confirmed based on visual inspection. The majority (> 70%) of mitochondria in a cell appeared tubular networks were defined as elongated, whereas the majority (> 70%) of mitochondria in a cell displayed short, round, fragmented were classified as fragmented. Mitochondria morphology was determined from ≥ 100 cells (per dish) of three different experiments. The percentage of cells with fragmented mitochondria was calculated. Cell counting was performed by three blinded reviewers.

### Statistical analysis

Data are presented as mean ± SEM. One‐way ANOVA was performed followed by Bonferroni *post hoc* test. *P* < 0.05 was considered statistically significant.

## Results

### Hypoxia induces ERK phosphorylation and promotes mitochondrial fission in APPSwe/Ind HEK293 cells

**Fig. 1:**
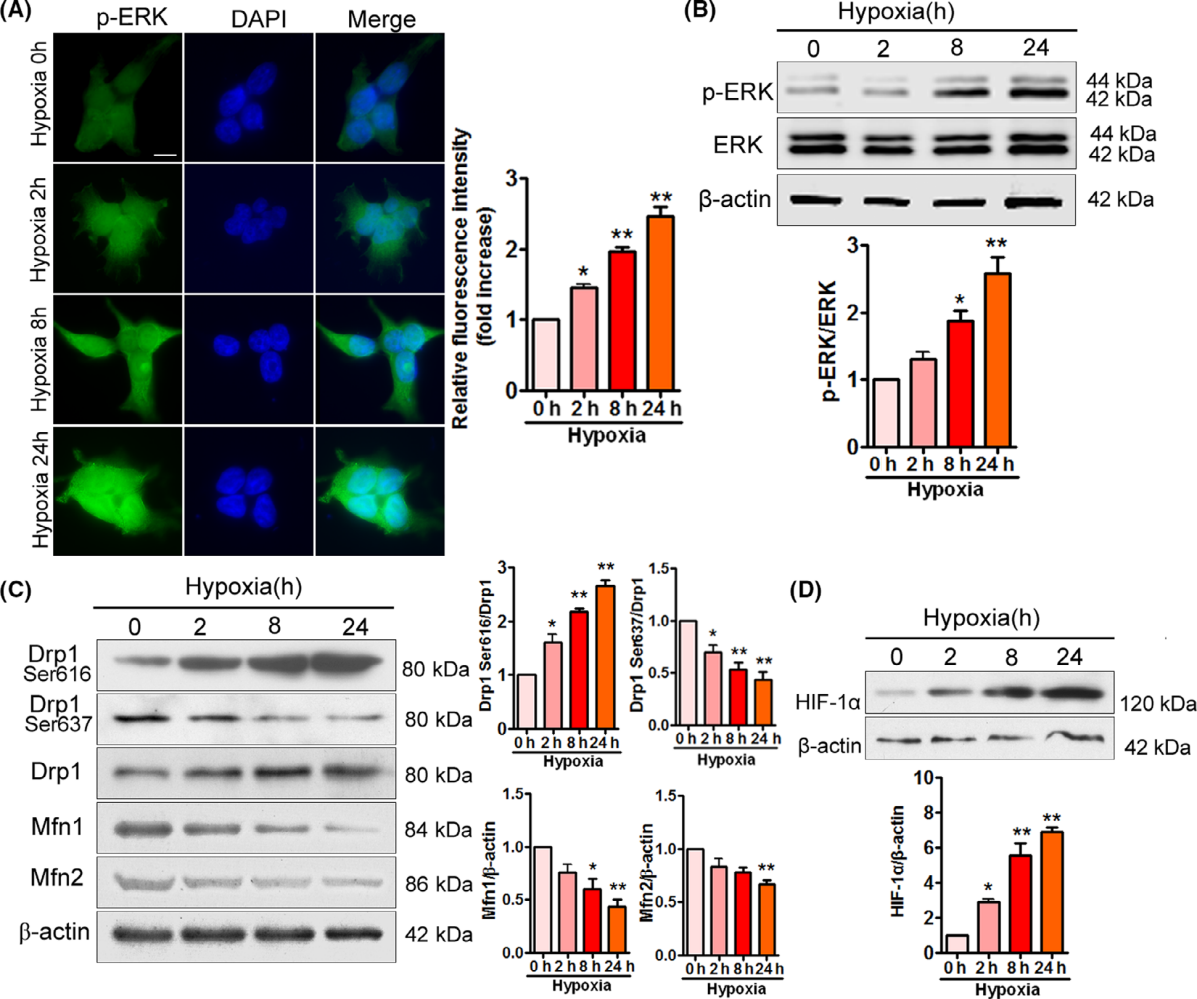
ERK phosphorylation and mitochondrial fission are increased in APPSwe/Ind HEK293 cells under hypoxia. (A) HEK293 cells were transfected with APPSwe/Ind plasmid and then exposed to hypoxia for the indicated times. At 48 h post‐transfection, cells were labeled with anti‐p‐ERK (green) antibody and nuclei were stained with DAPI (blue). Scale bars, 20 μm. Immunofluorescence was assessed from 100 cells of three different experiments. Image intensity was quantified using NIH imagej software (Bethesda, MD, USA). Data are presented as mean ± SEM. *P < 0.05 versus 0 h; **P < 0.01 versus 0 h. (B) Representative immunoblots and relative densitometric analysis for p‐ERK in APPSwe/Ind HEK293 cells under hypoxia. p‐ERK was normalized to total ERK. Data represent mean ± SEM of three independent experiments. *P < 0.05 versus 0 h; **P < 0.01 versus 0 h. (C) Cell lysates were subjected to immunoblot analysis using the indicated antibodies. Band density values of Drp1 (Ser616) and Drp1 (Ser637) were normalized to Drp1, whereas band density values of Mfn1 and Mfn2 were normalized to β‐actin. Data represent mean ± SEM of three independent experiments. *P < 0.05 versus 0 h; **P < 0.01 versus 0 h. (D) Cell lysates were probed with hypoxia‐induced factor‐1α (HIF‐1α). Data represent mean ± SEM of three independent experiments. *P < 0.05 versus 0 h; **P < 0.01 versus 0 h. Statistical significance was determined using one‐way ANOVA followed by Bonferroni *post hoc*.

Abnormal accumulation of p‐ERK is found in AD brains [23]. In addition, ERK is activated in response to hypoxia [27, 28, 36], which has been implicated in AD. Therefore, p‐ERK immunofluorescence staining was performed to observe ERK phosphorylation in APPSwe/Ind HEK293 cells exposed to hypoxia. As expected, hypoxia time‐dependently increased phosphorylation of ERK (Fig. 1A). We further analyzed total and phosphorylated ERK by immunoblotting. Hypoxia significantly elevated the phosphorylated to total ratio of ERK in APPSwe/Ind HEK293 cells (Fig. 1B), demonstrating that hypoxia triggers activation of ERK signaling. Mitochondrial fission is mainly regulated by Drp1, whereas mitochondrial fusion is controlled by Mfn1 and Mfn2 [4, 5, 37].

We then investigated the effect of hypoxia on mitochondrial fission and fusion proteins in APPSwe/Ind HEK293 cells. Notably, we found that hypoxia not only increased phosphorylation of Drp1 (Ser616) but also inhibited phosphorylation of Drp1 (Ser637) in a time‐dependent manner (Fig. 1C). In contrast, hypoxia caused a significant decrease in the levels of Mfn1 and Mfn2 (Fig. 1C). Hypoxia‐induced factor‐1α, a key regulator of the mammalian response to hypoxia, was detected to confirm that hypoxia exposure induced specific changes (Fig. 1D) in the present study. These results suggest that hypoxia strikingly elevates ERK phosphorylation, promotes mitochondrial fission, and suppresses mitochondrial fusion in APPSwe/Ind HEK293 cells.

### Hypoxia impairs mitochondrial function in APPSwe/Ind HEK293 cells

**Fig. 2:**
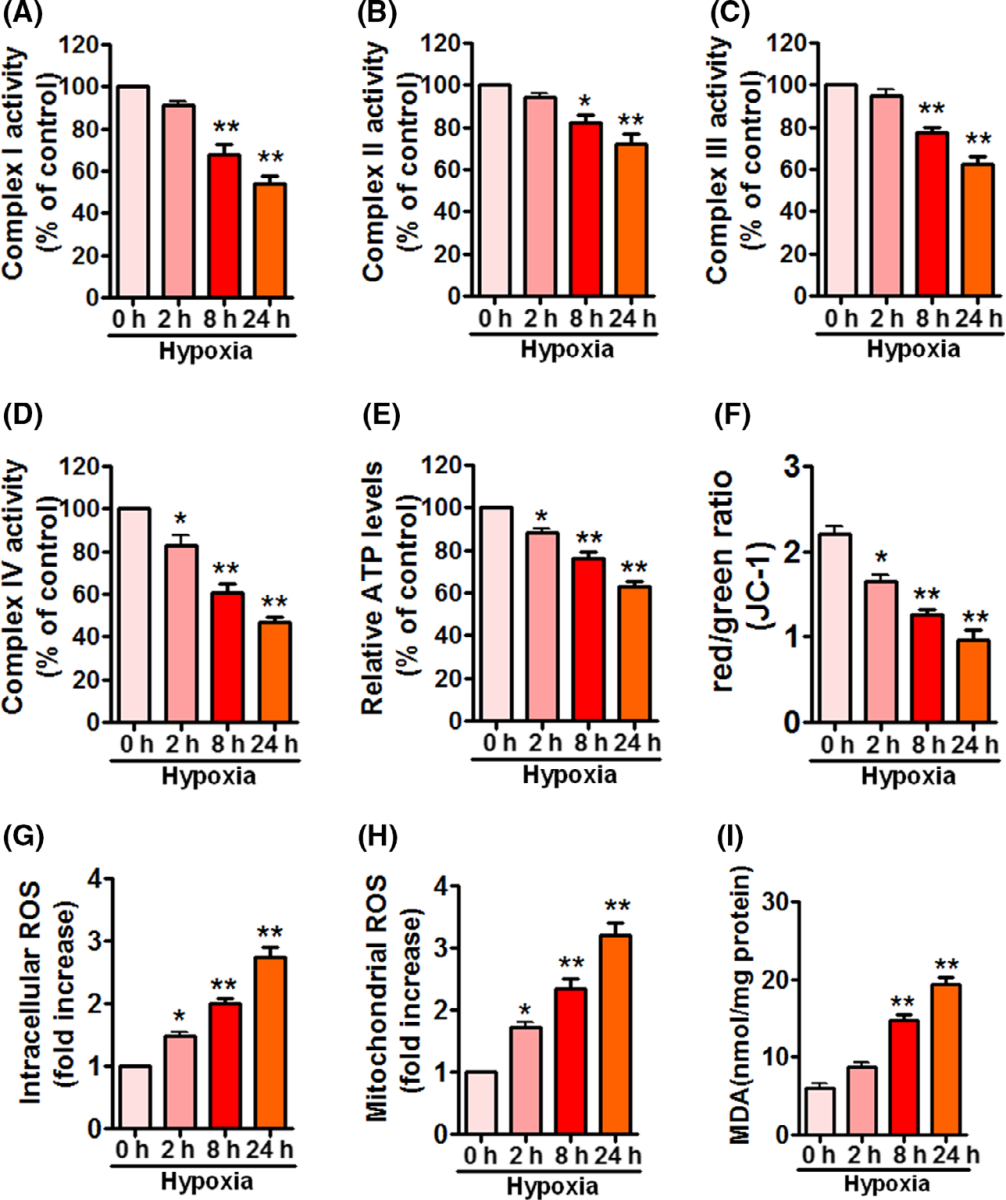
Hypoxia triggers mitochondrial dysfunction in APPSwe/Ind HEK293 cells. (A–I) HEK293 cells were transfected with APPSwe/Ind plasmid and then exposed to hypoxia for the indicated times. At 48 h post‐transfection, complex I activity (A), complex II activity (B), complex III activity (C), complex IV activity (D), ATP levels (E), Δψm (F), intracellular ROS (G), mitochondrial ROS (H), and MDA levels (I) were measured to monitor mitochondrial function and oxidative stress. Data represent mean ± SEM of three independent experiments. *P < 0.05 versus 0 h; **P < 0.01 versus 0 h. Statistical significance was determined using one‐way ANOVA followed by Bonferroni *post hoc*.

To investigated whether hypoxia induces mitochondrial dysfunction and oxidative stress in APPSwe/Ind HEK293 cells, we first measured activities of complexes I–IV in APPSwe/Ind HEK293 cells exposed to hypoxia. Hypoxia significantly reduced activities of complexes I–IV (Fig. 2A–D). Similarly, ATP levels were decreased under hypoxia (Fig. 2E) in a time‐dependent manner. To evaluate the effect of hypoxia on ∆ψm in APPSwe/Ind HEK293 cells, we monitored mitochondrial depolarization by JC‐1 staining. Hypoxia obviously diminished ∆ψm (Fig. 2F). These results suggest that hypoxia impairs mitochondrial function. Excessive mitochondrial fission is correlated with increased production of ROS. Therefore, we then observed intracellular and mitochondrial ROS levels by CM‐H2DCFDA and MitoSOX™ Red staining, respectively. Hypoxia time‐dependently elevated intracellular and mitochondrial ROS levels (Fig. 2G,H), confirming that hypoxia induces oxidative stress in APPSwe/Ind HEK293 cells. Additionally, we measured the effect of hypoxia on MDA release. Consistent with these observations, hypoxia significantly upregulated levels of MDA (Fig. 2I). These data demonstrate that hypoxia triggers mitochondrial dysfunction and oxidative stress in APPSwe/Ind HEK293 cells.

### Hypoxia enhances β‐ and γ‐secretase activities and facilitates Aβ generation in APPSwe/Ind HEK293 cells

**Fig. 3:**
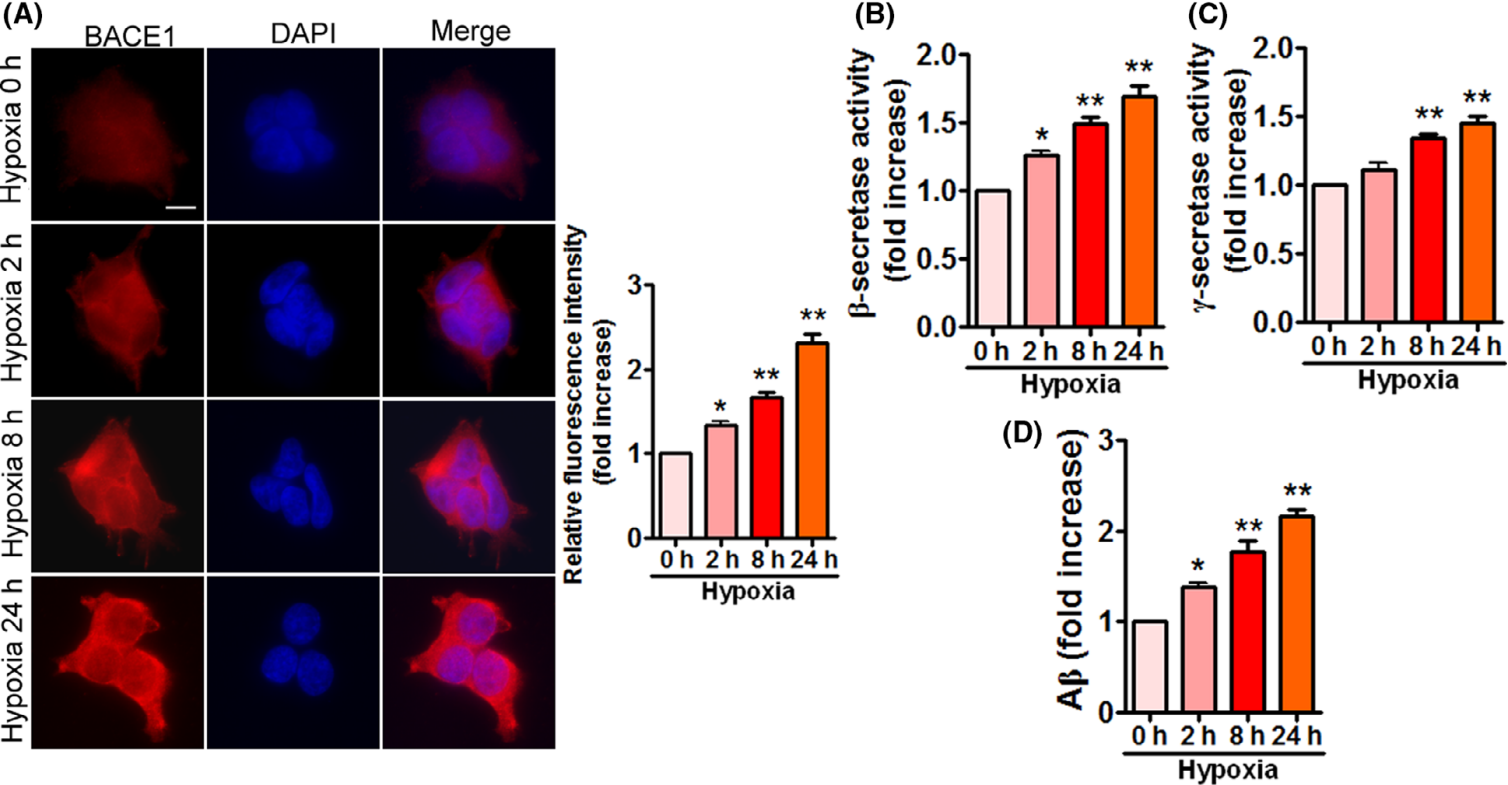
Enhanced BACE1 as well as γ‐secretase activities and Aβ_42_ accumulation are observed in APPSwe/Ind HEK293 cells under hypoxia. (A) HEK293 cells were transfected with APPSwe/Ind plasmid and then exposed to hypoxia for the indicated times. At 48 h post‐transfection, cells were stained with anti‐BACE1 (red) antibody and nuclei were stained with DAPI (blue). Scale bars, 20 μm. Immunofluorescence was assessed from 100 cells of three different experiments. Image intensity was quantified using NIH imagej software. Data are presented as mean ± SEM. *P < 0.05 versus 0 h; **P < 0.01 versus 0 h. (B–D) HEK293 cells were treated as indicated in (A). BACE1 activity (B), γ‐secretase activity (C), and Aβ_42_ production (D) were determined by ELISA. Data represent mean ± SEM of three independent experiments. *P < 0.05 versus 0 h; **P < 0.01 versus 0 h. Statistical significance was determined using one‐way ANOVA followed by Bonferroni *post hoc*.

BACE1 is the major β‐secretase responsible for Aβ production. Recent studies suggested that hypoxia increases BACE1 activity and Aβ accumulation [11, 13, 14, 15, 16]. On the other hand, hypoxia has been reported to upregulate γ‐secretase activity [15, 17]. Therefore, we examined BACE1 and γ‐secretase activities, and Aβ_42_ production in APPSwe/Ind HEK293 cells exposed to hypoxia. Hypoxia obviously enhanced BACE1 expression as well as activity (Fig. 3A,B), γ‐secretase activity (Fig. 3C), and Aβ_42_ production (Fig. 3D), indicating that hypoxia promotes Aβ accumulation by enhancing β‐ and γ‐secretase activities in APPSwe/Ind HEK293 cells.

### Hypoxia inhibits cell viability and stimulates cell death in APPSwe/Ind HEK293 cells

**Fig. 4:**
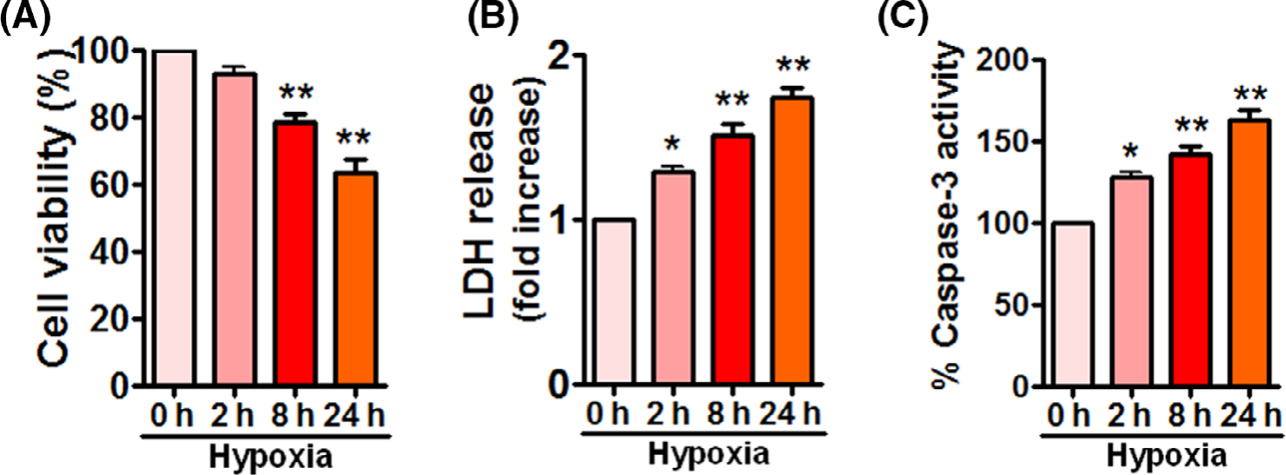
Hypoxia reduces cell viability and increases cell death in APPSwe/Ind HEK293 cells. (A–C) HEK293 cells were transfected with APPSwe/Ind plasmid and then exposed to hypoxia for the indicated times. At 48 h post‐transfection, cell viability and cell death were assessed using MTT assay (A), LDH release (B), and caspase‐3 activity (C), respectively. Data are presented as mean ± SEM of three independent experiments. *P < 0.05 versus 0 h; **P < 0.01 versus 0 h. Statistical significance was determined using one‐way ANOVA followed by Bonferroni *post hoc*.

To determine the effects of hypoxia on cell viability and cell death in APPSwe/Ind HEK293 cells, cell viability and cell death were assessed by MTT assay, LDH release, and caspase‐3 activity, respectively. As expected, hypoxia reduced cell viability (Fig. 4A). Hypoxia did not decrease cell viability at 2 h; however, it caused a significant decrease in cell viability at 8 and 24 h. In line with the result of cell viability, hypoxia dramatically induced LDH release (Fig. 4B) and increased caspase‐3 activity (Fig. 4C), indicating that hypoxia triggers cell death in APPSwe/Ind HEK293 cells.

### ERK inhibitor PD325901 and Drp1 inhibitor Mdivi‐1 attenuate hypoxia‐induced mitochondrial fission in APPSwe/Ind HEK293 cells

**Fig. 5:**
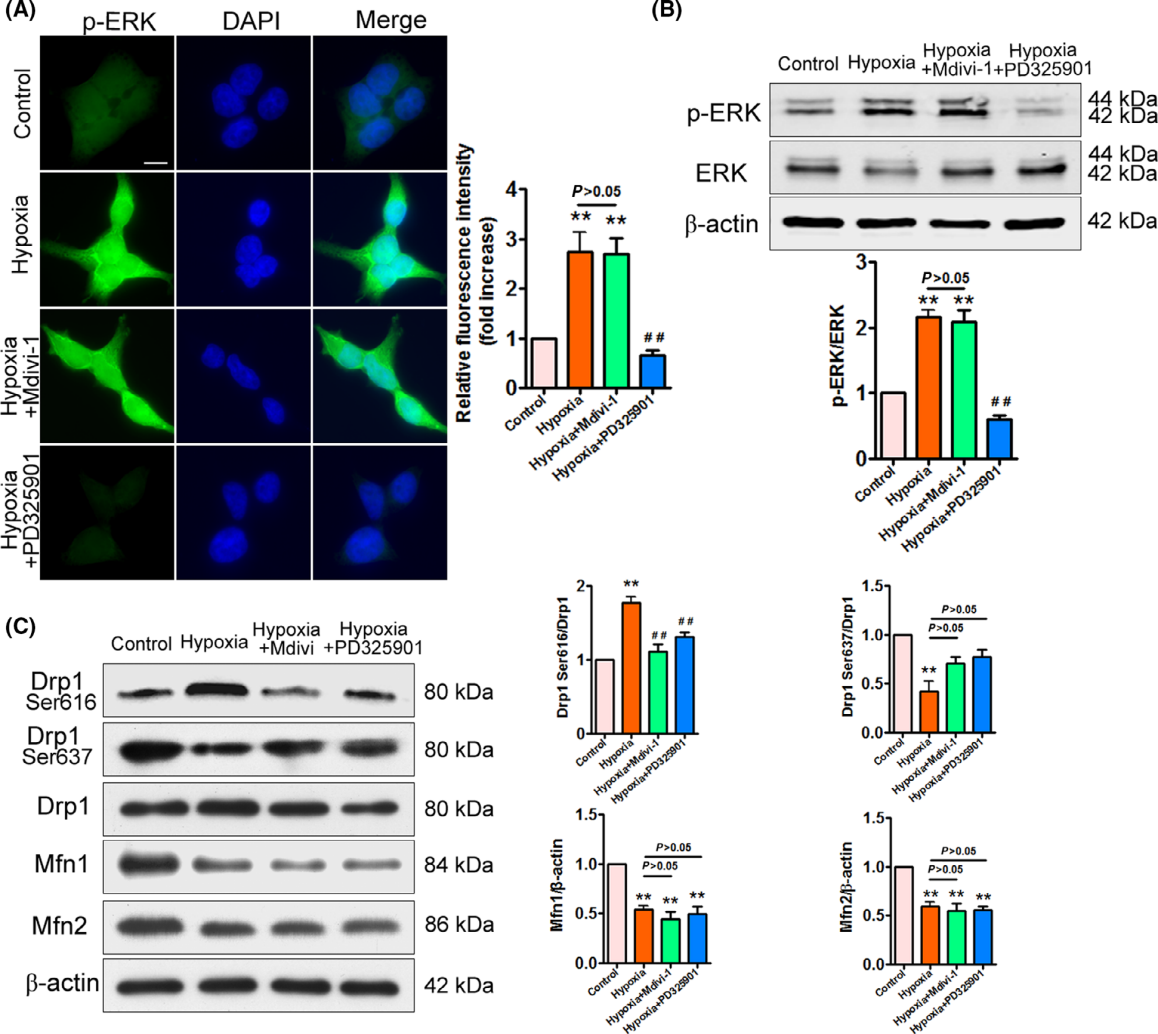
PD325901 and Mdivi‐1 regulate Drp1 phosphorylation in APPSwe/Ind HEK293 cells exposed to hypoxia. (A) APPSwe/Ind‐transfected HEK293 cells were exposed to hypoxia for 24 h, or pretreated with ERK inhibitor PD325901 (10 µm) or Drp1 inhibitor Mdivi‐1 (10 µm) for 1 h and then exposed to hypoxia for 24 h. At 48 h post‐transfection, cells were labeled with anti‐p‐ERK (green) antibody and nuclei were stained with DAPI (blue). Scale bars, 20 μm. Immunofluorescence was assessed from 100 cells of three different experiments. Image intensity was quantified using NIH imagej software. Data are presented as mean ± SEM. **P < 0.01 versus control; ^##^ *P* < 0.01 versus hypoxia. (B) Representative immunoblots and relative densitometric analysis for p‐ERK in APPSwe/Ind HEK293 cells treated as in (A). p‐ERK was normalized to total ERK. Data represent mean ± SEM of three independent experiments. **P < 0.01 versus control; ^##^ *P* < 0.01 versus hypoxia. (C) HEK293 cells were treated as indicated in (A). At 48 h post‐transfection, cell lysates were subjected to immunoblot analysis using the indicated antibodies. Band density values of Drp1 (Ser616) and Drp1 (Ser637) were normalized to Drp1, whereas band density values of Mfn1 and Mfn2 were normalized to β‐actin. Data represent mean ± SEM of three independent experiments. **P < 0.01 versus control; ^##^ *P* < 0.01 versus hypoxia. Statistical significance was determined using one‐way ANOVA followed by Bonferroni *post hoc*.

There is emerging evidence that inhibition of ERK exerts protective effects in AD [38, 39, 40]. Moreover, inhibition of Drp1 significantly reduces mitochondrial fragmentation in mouse models of AD [41, 42]. Thus, we examined the effects of ERK inhibitor PD325901 (10 µm) and Drp1 inhibitor Mdivi‐1 (10 µm) on ERK phosphorylation, mitochondrial fission, and fusion proteins in APPSwe/Ind HEK293 cells exposed to hypoxia. Immunofluorescence staining using p‐ERK antibody showed that hypoxia‐elevated ERK phosphorylation was inhibited by PD325901, whereas Mdivi‐1 failed to alter ERK phosphorylation in response to hypoxia (Fig. 5A). Furthermore, immunoblotting confirmed that PD325901 attenuated hypoxia‐induced ERK phosphorylation (Fig. 5B). By contrast, the ratio of phosphorylated to total ERK increased by hypoxia was unchanged in the presence of Mdivi‐1. Notably, following hypoxia treatment, PD325901 and Mdivi‐1 abolished phosphorylation of Drp1 (Ser616) without altering phosphorylation of Drp1 (Ser637) as well as levels of Mfn1 and Mfn2 (Fig. 5C). These data suggest that ERK inhibitor PD325901 and Drp1 inhibitor Mdivi‐1 alleviate hypoxia‐induced mitochondrial fission in APPSwe/Ind HEK293 cells and ERK‐Drp1 pathway is involved in the regulation of mitochondrial fission under hypoxia.

### PD325901 and Mdivi‐1 reverse hypoxia‐induced mitochondrial dysfunction and fragmentation in APPSwe/Ind HEK293 cells

**Fig. 6:**
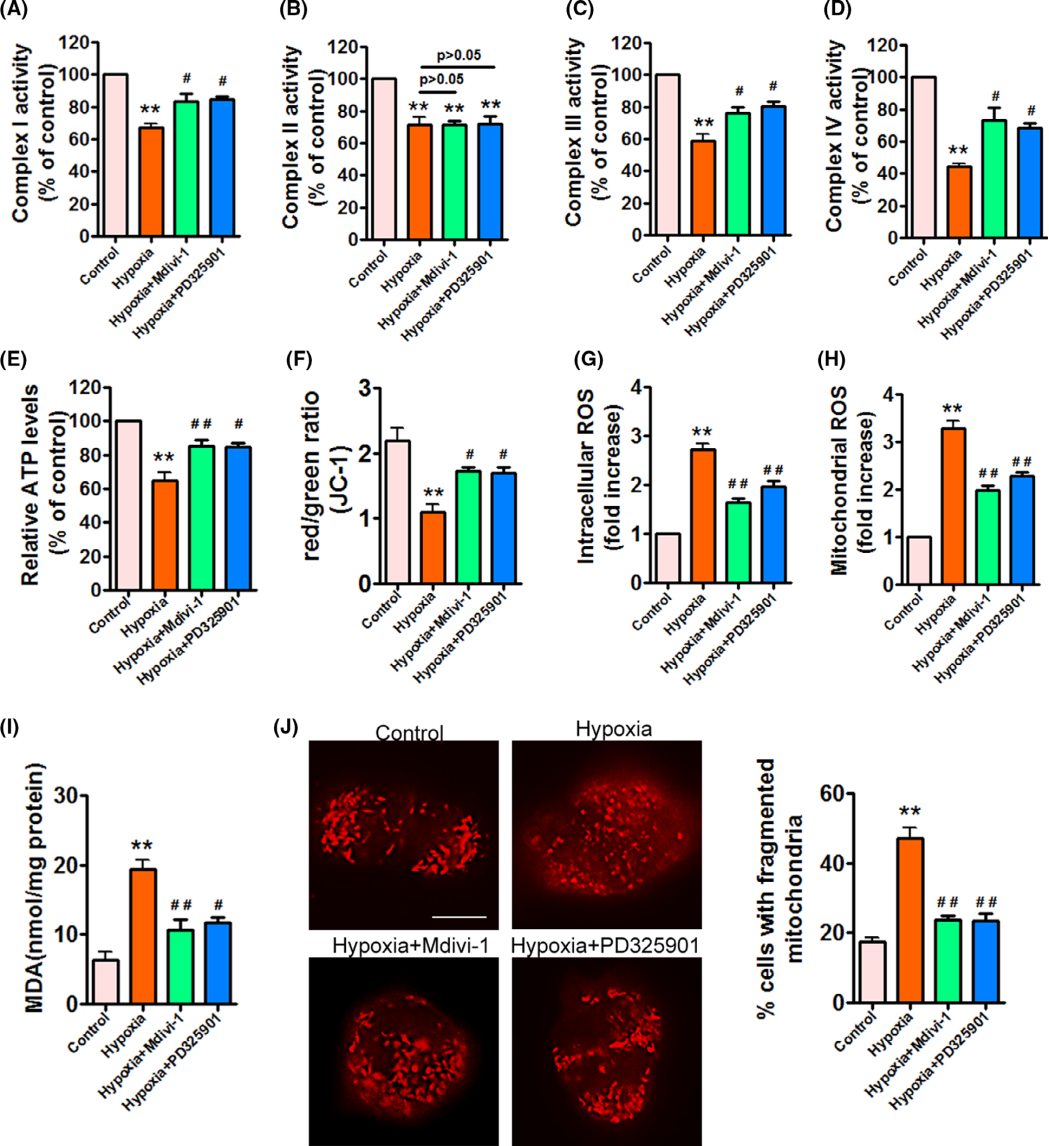
Hypoxia‐induced mitochondrial dysfunction and fragmentation are attenuated by PD325901 and Mdivi‐1 in APPSwe/Ind HEK293 cells. (A–I) APPSwe/Ind‐transfected HEK293 cells were exposed to hypoxia for 24 h, or pretreated with ERK inhibitor PD325901 (10 µm) or Drp1 inhibitor Mdivi‐1 (10 µm) for 1 h and then exposed to hypoxia for 24 h. At 48 h post‐transfection, complex I activity (A), complex II activity (B), complex III activity (C), complex IV activity (D), ATP levels (E), Δψm (F), intracellular ROS (G), mitochondrial ROS (H), and MDA levels (I) were measured to monitor mitochondrial function and oxidative stress. Data represent mean ± SEM of three independent experiments. **P < 0.01 versus control; ^#^ *P* < 0.05 versus hypoxia; ^##^ *P* < 0.01 versus hypoxia. (J) HEK293 cells were treated as indicated in (A). At 48 h post‐transfection, cells were labeled with MitoTracker Red to show mitochondria. Scale bars, 20 μm. Mitochondria morphology was assessed from 100 cells of three different experiments. Quantification is shown for percentage of cells with fragmented mitochondria. Data are presented as mean ± SEM. **P < 0.01 versus control; ^##^ *P* < 0.01 versus hypoxia. Statistical significance was determined using one‐way ANOVA followed by Bonferroni *post hoc*.

To investigate whether PD325901 and Mdivi‐1 would reverse hypoxia‐induced mitochondrial dysfunction, we measured mitochondrial function and oxidative stress in APPSwe/Ind HEK293 cells. PD325901 and Mdivi‐1 significantly attenuated hypoxia‐induced inhibition of complexes I, III, and IV activities (Fig. 6A,C,D) without affecting complex II activity (Fig. 6B). Moreover, PD325901 and Mdivi‐1 obviously increased ATP levels (Fig. 6E) and elevated ∆ψm under hypoxia (Fig. 6F). In addition, PD325901 and Mdivi‐1 evidently suppressed hypoxia‐induced intracellular (Fig. 6G) and mitochondrial ROS accumulation (Fig. 6H). Similarly, hypoxia‐induced MDA release was significantly decreased by PD325901 and Mdivi‐1 (Fig. 6I). Besides, mitochondria exposed to hypoxia appeared short and round shape, demonstrating that hypoxia triggers mitochondrial fragmentation. However, PD325901 and Mdivi‐1 remarkably diminished hypoxia‐induced mitochondrial fragmentation (Fig. 6J). Taken together, these data indicate that PD325901 and Mdivi‐1 reverse hypoxia‐induced mitochondrial dysfunction and oxidative stress in APPSwe/Ind HEK293 cells.

### PD325901 and Mdivi‐1 attenuate hypoxia‐increased BACE1 activity and Aβ_42_ generation in APPSwe/Ind HEK293 cells

**Fig. 7:**
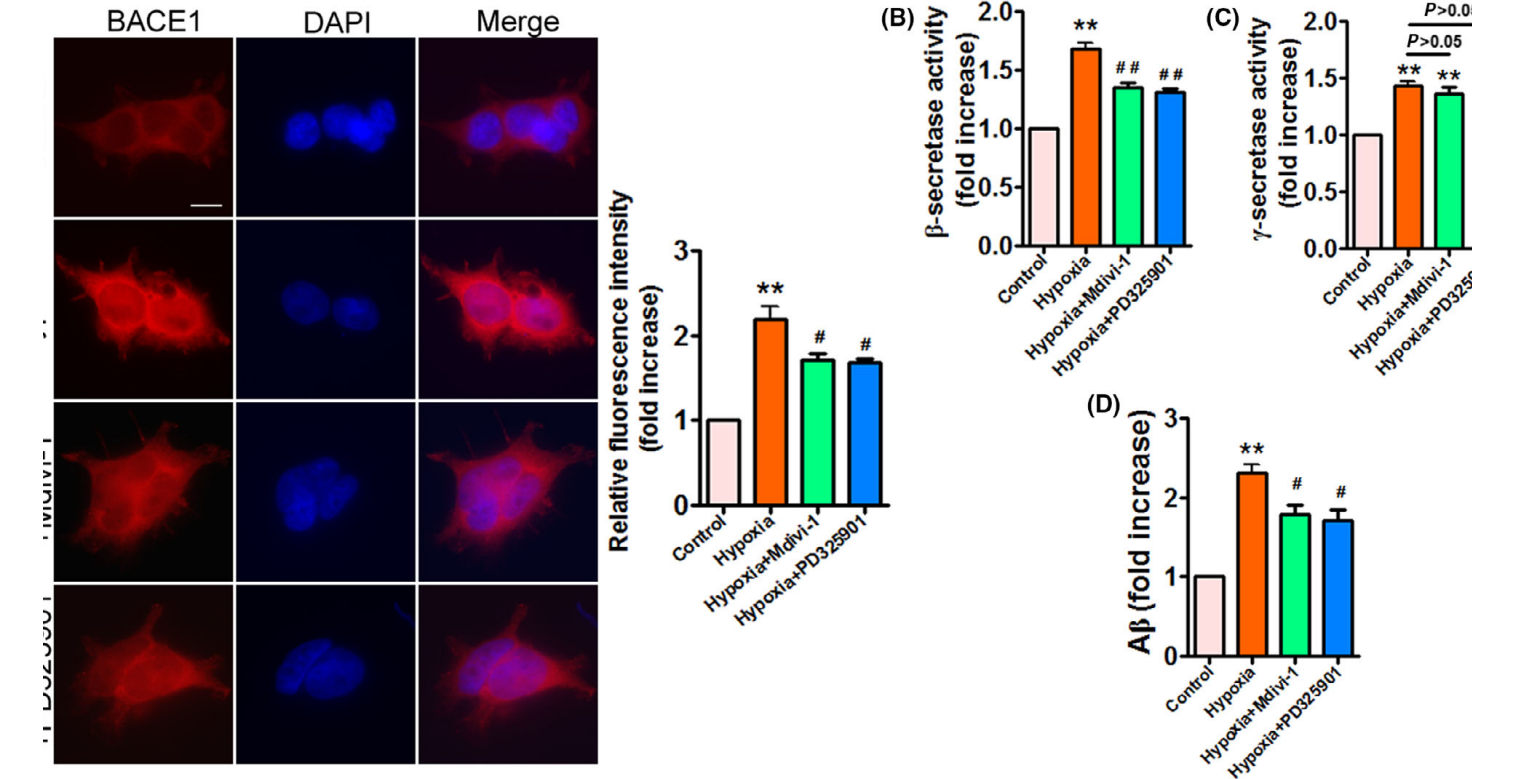
Hypoxia‐increased BACE1 activity and Aβ_42_ generation are inhibited by PD325901 and Mdivi‐1 in APPSwe/Ind HEK293 cells. (A) APPSwe/Ind‐transfected HEK293 cells were exposed to hypoxia for 24 h, or pretreated with ERK inhibitor PD325901 (10 µm) or Drp1 inhibitor Mdivi‐1 (10 µm) for 1 h and then exposed to hypoxia for 24 h. At 48 h post‐transfection, cells were stained with anti‐BACE1 (red) antibody and nuclei were stained with DAPI (blue). Scale bars, 20 μm. Immunofluorescence was assessed from 100 cells of three different experiments. Image intensity was quantified using NIH imagej software. Data are presented as mean ± SEM. **P < 0.01 versus control; ^#^ *P* < 0.05 versus hypoxia. (B–D) HEK293 cells were treated as indicated in (A). After 48 h post‐transfection, BACE1 activity (B), γ‐secretase activity (C), and Aβ_42_ production (D) were determined by ELISA. Data represent mean ± SEM of three independent experiments. **P < 0.01 versus control; ^#^ *P* < 0.05 versus hypoxia; ^##^ *P* < 0.01 versus hypoxia. Statistical significance was determined using one‐way ANOVA followed by Bonferroni *post hoc*.

We subsequently detected the effects of PD325901 and Mdivi‐1 on β‐ and γ‐secretase activities in APPSwe/Ind HEK293 cells exposed to hypoxia. Our results showed that hypoxia‐triggered high BACE1 expression and activity were obviously blocked by PD325901 and Mdivi‐1 (Fig. 7A–C). However, PD325901 and Mdivi‐1 had no effect on γ‐secretase activity under hypoxia. Moreover, PD325901 and Mdivi‐1 significantly suppressed hypoxia‐induced Aβ_42_ accumulation in APPSwe/Ind HEK293 cells (Fig. 7D). These results suggest that PD325901 and Mdivi‐1 attenuate hypoxia‐induced Aβ generation in APPSwe/Ind HEK293 cells through the inhibition of BACE1.

### PD325901 and Mdivi‐1 reduce hypoxia‐induced cell death in APPSwe/Ind HEK293 cells

**Fig. 8:**
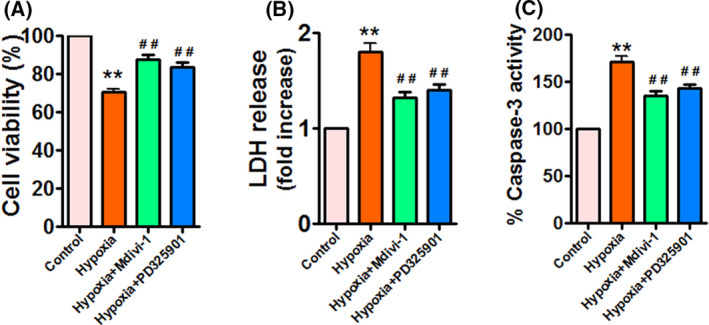
PD325901 and Mdivi‐1 reverse hypoxia‐induced cell death in APPSwe/Ind HEK293 cells. (A–C) APPSwe/Ind‐transfected HEK293 cells were exposed to hypoxia for 24 h, or pretreated with ERK inhibitor PD325901 (10 µm) or Drp1 inhibitor Mdivi‐1 (10 µm) for 1 h and then exposed to hypoxia for 24 h. At 48 h post‐transfection, cell viability and cell death were assessed using MTT assay (A), LDH release (B), and caspase‐3 activity (C), respectively. Data are presented as mean ± SEM of three independent experiments. **P < 0.01 versus control; ^##^ *P* < 0.01 versus hypoxia. Statistical significance was determined using one‐way ANOVA followed by Bonferroni *post hoc*.

Finally, we measured the effects of PD325901 and Mdivi‐1 on cell viability and cell death in APPSwe/Ind HEK293 cells under hypoxia. On the one hand, PD325901 and Mdivi‐1 significantly improved hypoxia‐reduced cell viability (Fig. 8A). On the other hand, PD325901 and Mdivi‐1 obviously ameliorated hypoxia‐triggered LDH release (Fig. 8B) and caspase‐3 activation (Fig. 8C), demonstrating that PD325901 and Mdivi‐1 inhibit hypoxia‐induced cell death. These findings show that PD325901 and Mdivi‐1 exert beneficial effects in APPSwe/Ind HEK293 cells exposed to hypoxia.

## Discussion

Hypoxia is a risk factor for AD or vascular dementia [11]. Increasing evidence reveals that hypoxia increases β‐ and γ‐secretase activities, leading to Aβ accumulation in brain [11, 14, 15, 16, 17]. Additionally, hypoxia has been reported to activate ERK signaling [27, 28, 43]. Indeed, ERK signaling pathway plays a key role in regulating synaptic plasticity and memory formation. Abundant studies suggested that inhibition of ERK exerts neuroprotective effects in AD. The activation of ERK by a‐1‐antichymotrypsin induces cell death and tau phosphorylation in cortical neurons [44]. Diammonium glycyrrhizinate reduces Aβ‐induced inflammation and memory deficits through inhibition of MEK/ERK pathway in an Aβ‐injected animal model [38]. Inhibition of ERK by thiacremonone attenuates cognitive impairments in APP/PS1 transgenic mice [39]. Besides, inhibition of ERK attenuates abnormal mitochondrial dynamics and rescues mitochondrial dysfunction in AD cybrid cell [40]. Importantly, the activation of ERK signaling contributes to phosphorylation of Drp1 at serine 616 and promotes mitochondrial fission [25, 26]. Hypoxia has been shown to stimulate mitochondrial fission, leading to dysfunctional and damaged mitochondria. Indeed, increased mitochondrial fission is associated with AD pathogenesis. However, pharmacological inhibition of Drp1 ameliorates mitochondrial fission, mitochondrial dysfunction, and Aβ deposition in AD animal model [41, 45]. Importantly, inhibition of Drp1 by Mdivi‐1 obviously reverses cognitive deficits in AD mice [41, 45], indicating that inhibition of hypoxia‐induced mitochondrial fission seems to be a good strategy to fight progression in AD. However, the relationship between hypoxia‐induced mitochondrial fission and Aβ accumulation associated with AD has not yet been identified.

In the current study, we show that ERK and Drp1 are involved in the regulation of mitochondrial fission and Aβ generation in APPSwe/Ind HEK293 cells exposed to hypoxia. Firstly, we found that ERK phosphorylation at Thr202/Tyr204 was significantly increased under hypoxia. Moreover, phosphorylation of Drp1 at serine 616 increases mitochondrial fission, whereas phosphorylation of Drp1 at serine 637 reduces mitochondrial fission. Therefore, we examined the effects of hypoxia on phosphorylated Drp1 levels in APPSwe/Ind HEK293 cells. Hypoxia remarkably enhanced phosphorylation of Drp1 at serine 616 and inhibited phosphorylation of Drp1 at serine 637. Conversely, mitochondrial fusion mediators Mfn1 and Mfn2 were significantly reduced in APPSwe/Ind HEK293 cells exposed to hypoxia. These data confirm that hypoxia induces activation of ERK kinase and regulates Drp1 phosphorylation, resulting in enhanced mitochondrial fission.

Mitochondrial fission is closely connected to mitochondrial dysfunction. Increasing evidence suggests that mitochondrial dysfunction including reduced mitochondrial membrane potential (∆ψm), dysfunctional mitochondrial axonal transport, and increased ROS is present in AD [6, 7, 46, 47, 48, 49]. Indeed, impaired mitochondrial respiratory chain complexes are also implicated in the pathogenesis of AD. Complex I activity is reduced in the brain of transgenic AD mouse model [50], whereas complex II activity is enhanced in APP/PS1 Mice [1]. Besides, a decline in complex III activity is found in Aβ_42_‐treated SY5Y cells [32]. Moreover, complex IV expression is decreased in a mouse model of AD [51]. Importantly, decreased activities of complexes III and IV are related to the accumulation of Aβ in mitochondria [52]. Therefore, we then investigated the effect of hypoxia on mitochondrial function. In line with enhanced mitochondrial fission, hypoxia significantly decreased activities of complexes I‐IV, induced ATP deletion, and reduced ∆ψm. Moreover, intracellular and mitochondrial ROS levels were dramatically elevated in response to hypoxia. Consistent with these observations, hypoxia increased the levels of MDA. These results indicate that hypoxia impairs mitochondrial function and promotes oxidative stress in APPSwe/Ind HEK293 cells. Hypoxia has been shown to regulate Aβ production associated with AD pathogenesis. Similarly, we found that hypoxia strongly promoted Aβ_42_ accumulation by increasing β‐ and γ‐secretase activities, demonstrating that hypoxia facilitates Aβ generation in APPSwe/Ind HEK293 cells. Subsequently, we investigated the effects of hypoxia on cell viability and cell death. Hypoxia dramatically ameliorated cell viability and induced cell death in APPSwe/Ind HEK293 cells. Taken together, these data indicate that hypoxia promotes mitochondrial fission, mitochondrial dysfunction, Aβ accumulation, and cell death in APPSwe/Ind HEK293 cells.

We next examined whether pharmacological inhibition of ERK or Drp1 decreases hypoxia‐induced mitochondrial fission. ERK inhibitor PD325901 obviously suppressed ERK phosphorylation enhanced by hypoxia, whereas hypoxia‐induced ERK phosphorylation was unchanged in the presence of Drp1 inhibitor Mdivi‐1. Interestingly, both PD325901 and Mdivi‐1 significantly inhibited phosphorylation of Drp1 (Ser616) without affecting phosphorylation of Drp1 (Ser637). However, PD325901 and Mdivi‐1 did not alter decreased levels of Mfn1 and Mfn2 induced by hypoxia. Importantly, Mdivi‐1 failed to diminish hypoxia‐induced ERK phosphorylation, supporting that ERK may serve as the upstream regulator of Drp1.

Taken together, these data suggest that ERK inhibitor PD325901 and Drp1 inhibitor Mdivi‐1 attenuate hypoxia‐induced mitochondrial fission through regulation of Drp1 phosphorylation without altering Mfn1 and Mfn2. Excessive mitochondrial fission is correlated with impaired mitochondrial function, we next examined the effects of PD325901 and Mdivi‐1 on hypoxia‐triggered mitochondrial dysfunction. PD325901 and Mdivi‐1 significantly abolished hypoxia‐triggered mitochondrial dysfunction, which was confirmed by enhanced activities of complexes I, III, and IV, increased ATP levels, elevated ∆ψm, decreased intracellular as well as mitochondrial ROS production, and reduced MDA levels. Besides, hypoxia‐induced mitochondrial fragmentation was remarkably diminished by PD325901 and Mdivi‐1, supporting that inhibition of mitochondrial fission prevents mitochondrial fragmentation under hypoxia. Furthermore, PD325901 and Mdivi‐1 significantly suppressed hypoxia‐induced high BACE1 expression and activity, resulting in reduced Aβ_42_ production in APPSwe/Ind HEK293 cells. These results provide evidence for the involvement of ERK/Drp1 signaling pathway in the regulation of mitochondrial fission and Aβ accumulation under hypoxia. Finally, we found that PD325901 and Mdivi‐1 protected APPSwe/Ind HEK293 cells against hypoxia‐induced cell death, suggesting that PD325901 and Mdivi‐1 exert neuroprotective effects in APPSwe/Ind HEK293 cells exposed to hypoxia. However, we cannot rule out the involvement of other pathways that were not observed in this study. For example, Stat2 promotes the phosphorylation of Drp1 at serine 616 [53]. Besides, Ca^2+^/calmodulin‐dependent protein kinase Iα (CaMKIα) has been shown to phosphorylate Drp1 [54]. Future studies will explore other pathways that may play an important role in the regulation of hypoxia‐induced mitochondrial fission and Aβ accumulation.

**Fig. 9:**
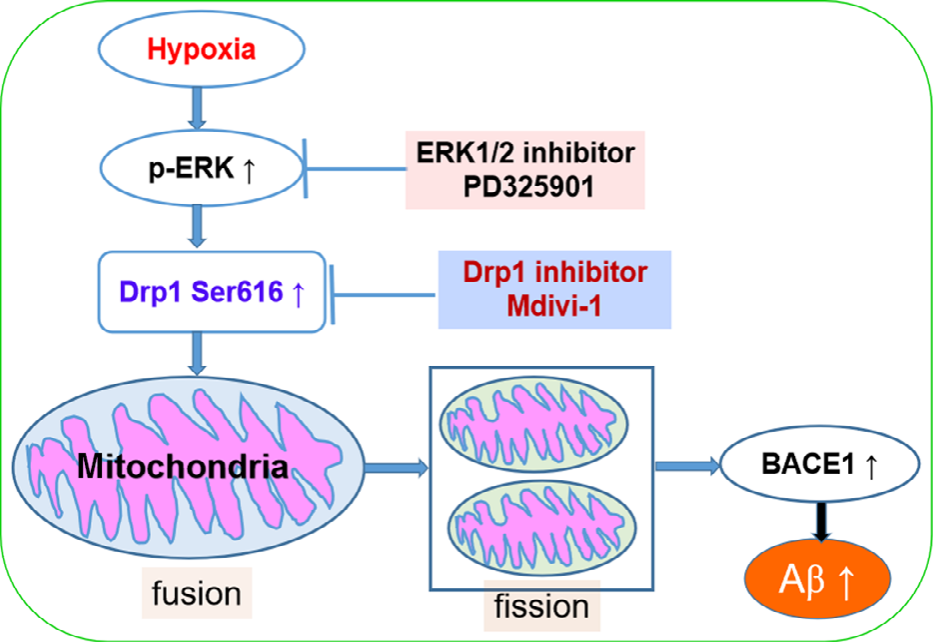
Schematic representation of possible mechanisms underlying hypoxia‐induced mitochondrial fission and Aβ generation in APPSwe/Ind HEK293 cells.

Collectively, ERK/Drp1 signaling pathway is partly involved in the regulation of hypoxia‐induced mitochondrial fission and Aβ accumulation. Pharmacological inhibition of ERK or Drp1 prevents mitochondrial fission and BACE1 activation, leading to the inhibition of Aβ generation in APPSwe/Ind HEK293 cells under hypoxia (Fig. 9). Controlling ERK/DRP1 signaling pathway under hypoxia may represent a potential therapeutic strategy against dementia associated with hypoxia, including AD.

## Conflict of interest

The authors declare no conflict of interest.

## Author contributions

YY and JC performed the experiments and analyzed the data. XG, JD and XX participated in interpretation and collection of data. HW designed the study and wrote the manuscript. YZ revised the manuscript.

## Data Availability

The datasets used in the current study are available from the corresponding author upon reasonable request.
